# Relationship between Training Load Management and Immunoglobulin A to Avoid Immunosuppression after Soccer Training and Competition: A Theoretical Framework Based on COVID-19 for Athletes’ Healthcare

**DOI:** 10.3390/healthcare9070856

**Published:** 2021-07-06

**Authors:** Markel Rico-González, José Pino-Ortega, Filipe Manuel Clemente, Naia Bustamante-Hernández

**Affiliations:** 1 Department of Physical Education and Sport, University of The Basque Country (UPV-EHU), 01007 Vitoria-Gasteiz, Spain; 2 BIOVETMED & SPORTSCI Research Group, University of Murcia, 30720 San Javier, Spain; josepinoortega@um.es; 3 Faculty of Sports Sciences, University of Murcia, 30720 San Javier, Spain; 4 Escola Superior Desporto e Lazer, Instituto Politécnico de Viana do Castelo, Rua Escola Industrial e Comercial de Nun’Álvares, 4900-347 Viana do Castelo, Portugal; 5 Instituto de Telecomunicações, Delegação da Covilhã, 1049-001 Lisboa, Portugal; 6 Department of Dental Medicine, Faculty of Medicine and Dentistry, University of Valencia, 46010 Valencia, Spain; naiabustamante@gmail.com

**Keywords:** immunology, IgA, SARS-CoV-2, pandemic, upper respiratory tract infection

## Abstract

Immunoglobulin A (IgA), which is the main effector against upper respiratory tract viruses such as severe acute respiratory syndrome coronavirus 2 (SARS-CoV-2), has been related to training load management. The aim of this systematic review was to establish the relationship between training load and salivary IgA based on current evidence in order to avoid immunosuppression after exercise and players´ vulnerability to virus contagion. A systematic review of relevant articles was carried out using two electronic databases (PubMed and Web of Science) until 19 May 2021. From a total of 127 studies initially found, 23 were included in the qualitative synthesis. These studies were clustered depending on stress level. The salivary IgA was analysed considering soccer-specific treadmill exercise and repeated sprint drills (*n* = 5), matches (*n* = 7), and during certain periods during the season or pre-season (*n* = 11). Repeated sprint ability tests and treadmill exercises are suitable exercises for the first steps on return to play periods yet still maintain social distance. A rest or moderate training sessions (technical/tactical) are suggested after official matches to ensure 16–18 h to recover IgA levels, while periods with multiple matches per week with limited recovery time should be avoided. Weekly training load should assume a small increment (<10%) to ensure IgA immune responses, especially, during the post coronavirus disease 2019 (COVID-19) season.

## 1. Introduction

In soccer, the quantification of training load is crucial to ensure players’ optimal performance, and to reduce injury occurrences [1]. Its quantification may be carried out through electronic performance and tracking systems or inertial devices, with additional technologies such as heart-rate devices, or using other strategies such as blood analyses or salivary tests. Each reports different information about players’ performance, and although some studies found a statistical relationship between them [2,3], it needs further research. Therefore, with the aim to detect issues related to biomarkers, blood or salivary test are needed. Among them, saliva samples, which make available hormone levels (e.g., testosterone and/or cortisol) and antibodies (e.g., immunoglobulin A), seem to be growing in popularity [4].

Antibodies are proteins that the immune system makes to fight attacks by bacteria, viruses, and toxins. The mucosal surfaces are protected by a network of organized structures located in the gut, urogenital tract, oral cavity, and respiratory system, collectively known as the mucosal immune system [5]. The “first line of defense” of the mucosal immune system against pathogens is the production of secretory immunoglobulin A (IgA) [6]. IgA has the ability to neutralize various bacterial virulence factors, limit the adherence and agglutination of bacteria, and prevent the penetration of foreign agents through the mucosa [6]. Specifically, IgA is the major effector of mucosal surface protection to inhibit the attachment of bacteria and viruses at mucosal epithelium in the mouth, throat, and upper respiratory tract, which limit upper respiratory (sinuses, nose, and throat) tract infections (URTI) [7,8,9]. In fact, a transient fall in salivary IgA (sIgA) was shown to be a good predictor for increased risk of URTI [8,9,10]. Nowadays, coronavirus disease 2019 (COVID-19) caused by severe acute respiratory syndrome coronavirus 2 (SARS-CoV-2) RNA in the upper respiratory tract has become the most popular URTI. Recently, the quick dissemination of this novel coronavirus to the rest of the world induced over 177 million confirmed cases worldwide and over 3.82 million deaths (up to May 2021), becoming the most important single event of the 21st century so far [11,12].

Since the IgA antibody has a relationship with training load, stress symptoms, and symptoms of upper respiratory tract infections [6,7,13], sIgA has become an important marker to extract information about training load and soccer players immune responses. In this regard, it showed acute episodes of moderate exercise have little impact on mucosal immunity, but, prolonged exercise and intense training could cause a decrease in sIgA [6,14,15,16]. The mechanisms underlying alterations in mucosal immunity with acute and chronic exercise are likely to be largely related to altered activity of the hypothalamic-pituitary-adrenal axis (i.e., activation of the sympathetic nervous system), with inhibitory effects on IgA synthesis and IgA transcytosis [6].

It is likely that some studies such as Padoan et al. [17], which through a longitudinal protocol found a relationship between decrement in sIgA and SARS-CoV-2, have encouraged some authors to make suggestions about training load management after confinement caused by the COVID-19 world pandemic [12,18]. These authors suggest very intense training loads could put the health of players at risk due to a lowered antibody response [12,18]. However, to decontextualize training recommendations on return to play after the COVID-19 pandemic, some authors encourage sharing information [19]. In fact, it may be more helpful to team staff and/or football federations to make decisions on return to play after the COVID-19 pandemic. A previous systematic review summarized the relationship between training load, IgA, and URTI in general team sports, which did not include soccer [20]. Therefore, the aim of this systematic review was to analyze the relationship between soccer training load and sIgA based on current evidence. It may be necessary to bridge the gap of previous narrative reviews about how stressors individually and collectively influence immunity [4].

## 2. Materials and Methods

The systematic review was reported in accordance with the guidelines for performing systematic reviews in sport sciences [21] and Preferred Reporting Items for Systematic Reviews and Meta Analyses (PRISMA) guidelines [22].

### 2.1. Design

A systematic search was performed by two authors (M.R.-G, N.B.-H.) to identify articles published before 19 May 2021 in two electronic databases (i.e., PubMed, and Web of Science). The authors of this review were not blinded to journal names or manuscript authors. The search was conducted throughout the full text. The search strategy combined terms covering the topics of (1) sport, (2) intervention related variables; (3) words related with expected outcomes. The keywords were connected with AND to combine the three groups and using OR to link the words of each group: (soccer OR football) AND (salivary) AND (immunity OR “Immunoglobulin A” OR sIgA OR “sIgA secretion rate” OR “srIgA” OR “mucosal immunity” OR “upper respiratory symptom *” OR URS). Due to the high number of articles found, the present systematic review summarized all articles performed in soccer, while the PART I was focused on the remaining team sports (rugby, Australian football, basketball, handball, ice hockey, futsal) [20].

### 2.2. Screening Strategy and Study Selection

After completion of the search, results were compared between researchers to ensure the same number of articles was found. Then, one of the authors (MR) downloaded the main data from the articles (title, authors, date, and database) to an Excel spread sheet (Microsoft Excel, Microsoft, Redmond, WA, USA) and removed duplicate records. Subsequently, the same authors screened the remaining records to verify the inclusion/exclusion criteria using a hierarchical approach in two phases. The papers were excluded when they met the following exclusion criteria: (1) articles not considered soccer athletes; (2) not salivary related interventions; and (3) biomarkers non-related with immunology (IgA); (4) articles that assessed the influence of another factor (e.g., nutritional intervention) in IgA response, or the effects of IgA in another contexts (e.g., oral health); (5) non-original research papers (i.e., systematic reviews, conferences or meetings).

### 2.3. Data Analysis

Each article was classified depending on the type of stressor: (1) soccer-specific treadmill and repeated sprint drills, (2) matches and, (3) season periods (any period during the season or pre-season that contains only training sessions or training sessions and matches). Each reference was detailed based on the following: sample, stressor factor and its characteristics, sIgA test moment and outcomes, relationship with URTI, and concluding remarks and lessons learned. The specifications about them were provided when the data of several studies were provided in the discussion and conclusions.

## 3. Results

### 3.1. Identification and Selection of Studies

**Figure 1:**
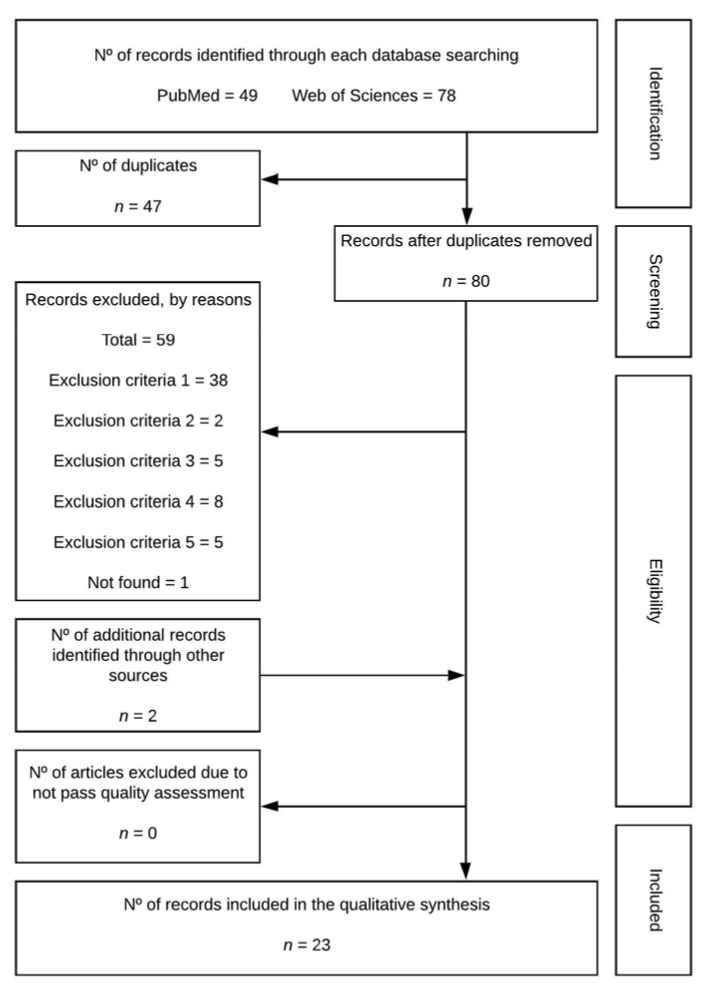
Flowchart.

A total of 127 (PubMed = 49; Web of Sciences = 78) documents were initially retrieved from the databases, of which 47 were duplicated. Thus, a total of 80 articles were downloaded. After screening the titles and abstracts against criteria 1 (where applicable), and the full text of the remaining papers against the same criteria, 38 studies were excluded. From the 42 articles, 2, 5, 8, and 5 were ruled out against criteria 2, 3, 4 and 5, respectively. Additionally, 2 articles were added from external sources. Therefore, 23 studies were included for the qualitative analysis (Figure 1).

### 3.2. Study Characteristics

#### 3.2.1. Repeated Sprint Ability and Treadmill Exercises

From the 23 articles, five pertain to soccer-specific training exercises. One pertains to a repeated sprint ability test [23], and four involved a soccer-specific treadmill protocol [24] composed of six 15-min periods with a 15 min rest time between the third and fourth periods [25,26,27,28]. Among these studies, neither non-professionals (*n* = 19) [27,28] or semi-professionals (participants = 10) [25], nor professional soccer players (participants = 40) [23,26] reported significant decrements in sIgA levels after repeated sprint ability test or treadmill exercises (Table 1).

#### 3.2.2. Matches during Intensive and Non-Intensive Fixture Schedules

From the remaining 18 articles, seven examined matches as a stressor [5,9,29,30,31,32,33]. Three of them included young players: two with U15 players (*n* = 42) [5,31], and one with U19 players (*n* = 14) [9]. The remaining four articles included female (players = 16) [29], and male (total players = 54) professional players [30,32,33]. Among the results, most articles reported decrements in IgA after soccer matches [5,9,30,31,32,33]. In addition, two found a relationship between IgA decrements and URTI [5,9]. However, three studies did not report a clear relationship from official [29,34] and non-official [5] matches. (Table 2).

#### 3.2.3. Preparation Periods (Matches/Training or Training) during Intensive and Non-Intensive Fixture Schedules

The remaining 11 articles considered match and training sessions as a stressor. From them, four articles collect data in a season without considering a specific period (e.g., microcycle) [8,13,16,35], one with two training and two matches [36]. The remaining articles entailed 4 non-consecutive [3] and consecutive [2] training sessions, and during a microcycle (i.e., training session -4, -3, -2, and -1) [34,37]. The main results highlighted a decrement in IgA protein level during competitive periods (including training sessions) [8,10,13]. Four articles found IgA decrements after non-consecutive [38] and consecutive [2,34,37] high intensity training session. Moreover, four articles found a relationship between IgA and URTI [8,9,10,35]. These studies employed professional male players (total players = 137) [2,3,34,36,37,38], young players (*n* = 26) [8], another with adolescents (i.e., U15, U17, and U19) [10], and the remaining three with male (*n* = 12) [35] and female (*n* = 26) [13,16] collegiate players. (Table 3).

Additionally, from 18 articles, nine considered periods with multiple matches per week with limited recovery time as a characteristic of stressor intervention: three of them during soccer-specific protocols [25,27,28], one during season periods [36], and five during consecutive matches [5,9,29,31,32] (Table 1, Table 2 and Table 3).

## 4. Discussion

The aim of this systematic review was to analyze the relationship between training load and sIgA based on current evidence. The main findings were: (1) sIgA is a valid metric to assess immune function of players; (2) repeated sprint ability test and treadmill exercises were not sufficient stressors to induce immune suppression sIgA protein response; (3) although it is not clear the impact of non-official matches and the periods with a unique match per week in IgA, the official matches and periods with multiple matches per week with limited recovery time induced decrements in IgA responses; (4) 18–36 h after a match may be needed to ensure a restart of IgA levels to a pre-match baseline; (5) weekly training load increases should not exceed the 5–10% with respect to the baseline of the previous training sessions; and, (6) IgA might be more responsive to training volume rather than intensity.

### 4.1. Repeated Sprint Ability and Treadmill Exercises

The main findings showed the sIgA test measured before, during, and after repeated sprint ability tests [23] or during treadmill soccer-specific protocols [25,26,27,28] did not compromise sIgA responses. Because soccer performance is sport that combine high-intensity efforts with lower intensities [40], seven 40 m sprints with 25 s rest seem similar to soccer-specific efforts. However, it did not suppress the immune system, likely due to a lack of real competition situations and psychological stressors [5,13,41]. Even intermittent exercise seems to induce lower suppression on IgA response than continuous exercise, even though the same treadmill work-rate was programmed [26]. This is consistent with Figueiredo et al. [2], who found IgA might be more responsive to volume (i.e., training time and distance covered) rather than intensity (distance covered per min, and high-speed running), as was supported by other studies [3]. Additionally, Moreira et al. [30] did not find a significant effect in sIgA even when the volume of the match was reduced. Therefore, this leads us to suggest increases in training volume rather than intensity must be considered with caution to avoid immunological responses from IgA protein.

In addition to the protocol proposed by Rodrigues de Araujo et al. [23], some studies that analyzed saliva sampled after a test showed high correlations with soccer-specific volume/intensity efforts [24]. The protocol was composed of two 45 min periods. Each “half” included seven static pauses (60 s, 30 s, and 15 s), 40 actions of walking (2 at 33 s, 2 at 30 s, 36 at 25 s), 30 jogging bouts (10 at 49 s, 20 at 26 s), 22 bouts cruising (22 at 12 s), and 16 sprints (2 at 9 s and 14 at 8 s). After the first 45 min and a 15 min intermission, the exercise was continued for a further 45 min to correspond to the duration of a soccer game [26]. However, this protocol neither found differences before in none of these scenarios: (1) during (i.e., in a rest time) and after (i.e., immediately, 6 h, 24 h or 48 h) congested fixtured treadmill exercises; (2) with two bouts and 2 h between them [27]; (3) with 2 bouts and 48 h rest between them [28], or, (4) with 3 bouts and 48 h rest between them [25]. Therefore, considering that 18–36 h have been needed to recover IgA levels [30,33,36], and analyzing that no effects in sIgA were found in two treadmill exercise separated by 2 h, strength and conditioning coaches and medical staffs may implement both of these protocols [23,24] on the first steps on return to play period. This suggestion should be followed, at least, when the social distancing is necessary [12,42,43,44], or during confinement periods. At a practical level, at least the repeated sprint ability test should be performed with no more than five players participating in training sessions at the same time, with 5 m separating the players, and with their own lane for running and sprints. If the requirement cannot be met, the same lane may be used by more players, but they must maintain a distance between each other of at least 40 m when sprinting [12].

### 4.2. Matches during Intensive and Non-Intensive Fixture Schedules

Although some authors did not find IgA decrements after soccer matches [29,34], most authors agree with a decrement in IgA levels (up to 75% from baseline) after soccer matches [5,9,30,31,32,33], which may induce URTI [5,9]. Thus, training load management may be crucial to avoid virus contagion. In fact, a low level of sIgA is associated with an increased risk of URTI in athletes [9,10], and SARS-CoV-2 [17]. In this sense, strength and conditioning coaches should programme 18–36 h of rest after a match [30,33,36] to ensure a restart of IgA level to a pre-match baseline. These rest days or days with moderate intensity training may be focused on the acquisition of technical/tactical concepts as programmed by Owen et al. [3].

In addition to acute exercise, it is well known that heavy chronic exercise is associated with an increased risk of IgA suppression and URTI [13,30]. In this regard, some studies found a sIgA decrease during periods with multiple matches per week with limited recovery time [9,31,32]. The accumulated fatigue induced by the physiological and psychological stressors imposed by training and competition in a short timeframe might negatively affect their mucosal immunity [9,31,32]. It is clearly a failure to recover fully between sessions was suggested to cause immunosuppression [28], the aforementioned 18–36 h recovery period is recommended [6,30,33,36]. Similarly, Page et al. [25] suggested players possess the capacity to complete two games with <72 h recovery, but that the risk of injury is increased if a third game is completed at this frequency [25]. Subsequently, soccer clubs and federations should be aware of the competition calendar programming as a protective action to minimize players’ vulnerability to contact SARS-CoV-2 or other viruses. However, those league competitions in which are often played a unique match per week may be started. As a practical alternative, squad rotation is an option where the playing roster is sufficiently large. Rather than excluding a player from a game to facilitate recovery, a substitution strategy may be more practical given the physical demands. Page et al. [25] found substitutions made no later than the 60^th^ minute of the match may have a beneficial effect on reducing the fatigue response associated with match-play. It is consistent with Morgans et al. [32] who found an evident declination in sIgA in players that played more than 50% of the total minutes played.

### 4.3. Preparation Periods (Matches/Training or Training) during Intensive and Non-Intensive Fixture Schedules

IgA protein levels tend to decrease during time periods with training, and even more so if these periods include high-intensity sessions [2,34,37], and during the competitive period [8,10,13]. In addition, despite one exception [16], different studies found relationships between IgA and URTI [8,9,10,35]. In fact, Putlur et al. [13] stated 82% of illnesses could be explained by a preceding decrease in sIgA. In practical terms, a decrement of non-common IgA levels could lead to an URTI three days later [35]. This finding enhances sIgA as a useful predictor for URTI.

Depending on the level of the competition and team, soccer players should be prepared to carry out 3–7 training sessions during domestic leagues, or tournament preparations. While some high-level soccer teams often compete twice a week, most young, amateur and the vast majority of professional soccer teams have to play one match a week [1]. A training week (i.e., microcycle) is typically designed to ensure readiness to play in the next official match. Regardless of the competition level and match features, the organization of the weekly training load should warrant peak performance in the most important session of the week: the official match [1]. From studies that analyzed the effects of IgA over four training sessions during the same week (match day -4, -3, -2, -1), the authors found the accumulation of training sessions induce detectable perturbations to mucosal immunity. Thereby it provides objective evidence for the administration of appropriate interventions to prepare players for the physical stress for the game day by self [2,34,37]. Therefore, the aforementioned suggestions about rest or moderate training days should be applicable to each microcycle.

In addition to the combination of rest days, high-intensity and moderate intensity sessions, a weekly training load increment that does not exceed 5–10% from the previous workload should be considered [4,43] during each microcycle. Gleeson [6] indicated moderate exercise can restore optimal antibody responses in the face of stressors. Therefore, strength and conditioning coaches should program a short prophylactic period (i.e., detraining period) after an intense session to attenuate mucosa immunosuppression related to URTI symptoms [8]. In addition, these suggestions should be completed with nutritional (e.g. carbohydrate enrichment diet) [18,44,45], lifestyle (e.g., sleep disturbance) [4,18,44], and other (e.g., adverse environment conditions, international travels, hygiene suggestions) complementary strategies [4].

In summary, since some authors suggested the management of training loads to avoid immunosuppression against the virus (e.g., SARS-CoV-2) contagion, strength and conditioning coaches and sports scientists are called to share information based on real data. In this sense, the present systematic review highlights that moderate (5–10%) increments of weekly volume/intensity training load during domestic leagues and, the periodization of rest and/or moderate training sessions after matches or very high-intensity efforts. In addition, since some authors encourage coaches to program training tasks with social distancing during the first periods of returning to play, repeated sprint ability tests and treadmill soccer-specific exercises may be suitable alternatives. However, they should be completed with additional tasks which address the limitations about non-real situations of these non-contextualized drills. Finally, taking to account the pressures derived from the economy, together with those of social origin, lead to an almost forced start of sports competitions after a period of confinement worldwide [19], the suggestion of suspense the periods with multiple matches per week with limited recovery time from calendars during the post-COVID-19 home-confinement soccer season is be made with caution. However, since older players (>15 years) showed stronger immune responders than young players [10], this suggestion could be followed, at least, in non-professional populations.

## 5. Study Limitations

Since economic, social, and health issues must be considered in decisions on competition restart and other federation concerns related to the pandemic, and considering that SARS-CoV-2 propagation depends on a wide range of factors in addition to those mentioned in this systematic review, the authors would only like to make some recommendations and best practices based on current evidence.

These concluding remarks may highlight some recommendations to prevent an athlete’s vulnerability to immunosuppression, which may be an important factor in their return to play post COVID-19. Therefore, the relationship between training load and sIgA is evidence, while the relationship between IgA and URTI is not clear. However, the relationship between these stressors and the risk of illness should be further researched. In this regard, the authors encourage practitioners to share daily information on the dynamic of training load during the previous 15–20 days prior to a contagion.

## 6. Conclusions

Lessons learned and concluding remarks are six-fold: (1) the salivary IgA test is a valid metric to the control immune function of players, at least, during the initial stages of return to play after the COVID-19 confinement period. (2) The repeated sprint ability test and treadmill exercises are suitable training protocols to perform during periods with social-distancing necessities (e.g., the first steps of return to play) or during confinement. (3) The impact of non-official matches and periods with a single match per week in IgA is not clear. Therefore, a restart of leagues in which a unique match per week is played, and considering social and economic pressures, may be suitable after the confinement period. However, we suggest a reduction of those competitions which induce a period with multiple matches per week with limited recovery time to avoid immunosuppression after exercise and players vulnerability to virus contagion. (4) To ensure a restart of IgA level to a pre-match baseline, programming 18–36 h after a match formed by a rest day and/or moderate intensity training sessions (i.e., technical/tactical sessions). (5) A progressive increment of weekly training load should be carefully undertaken (increment of 5–10% weekly) to attenuate immunosuppression by IgA protein. Finally, (6) although it is not clear, and further research is necessary to support this affirmation, it could be that sIgA might be more responsive to training volume (e.g., training time, the volume of training, time spent in competition) rather than intensity (distance covered per min, and high-speed running). Moreover, this affirmation should be supported with some of the wide possibilities of variables derived from the current technology.

## 7. Future Research

Interestingly, some articles suggested some concluding remarks that should be considered with caution due to the lack of further research. They are summarized in two ways: (1) the relationship between electronic performance and tracking systems, and subjective exertion and wellness questionnaires with IgA levels; and, (2) the relationship between other biomarkers and IgA.

*Technology and questionnaires, and IgA*: due to the fast training/competition evaluation and prescription that new technologies (i.e., electronic performance and tracking systems) allow, and considering that today there is not a professional soccer club without these essential tools for training load monitoring, future research should associate variables derived from global positioning systems, semi-automatic video camera systems or local positioning systems. This idea was started by authors who found that IgA levels are dependent on some global positioning system variables (total distance variable, accelerations, and total load) [2,3], RPE [3,9,30], and wellness questionnaires [37].

*Biomarkers and IgA*: since some studies observed soccer athletes with testosterone level decreases less than those for IgA [10,29,33], it was speculated increased testosterone concentration after football matches may play a protecting role against immune suppression usually observed after intense exercise.

## Figures and Tables

**Table 1:** Immunoglobulin A (IgA) responses considering repeated sprint ability and treadmill exercises as a stressor during intensive or non-intensive fixture schedules.

Ref.	Sample	Stressor				Immunology (IgA)		URTS/URTI Relationship	Lesson Learned and Concluding Remarks
		Int. Fix. Schedule	Test	Volume	Intensity	Salivary Test	Outcomes		
Rodrigues de Araujo et al. [23]	32 male professionals	No	HIIE [39]	7 × 40 m sprint with 25 s between each of them	Sprint	Post exercise (Immediately)	Unaltered in spite of its exhaustive characteristics	-	HIIE tests could be conducted without short-term immunosuppression risks
Sari-Sarraf et al. [26]	8 males not professionals	No	Soccer-specific intermittent [24] and continuous exercise	Intermittent exercise: 6 periods of 15 min. Continuous exercise: 2 periods of 45 min.	Exercise at the same average work-rate: Continuous: 141 HR/min; RPE: 10.8; Vel: 9.7 km/h. Intermittent: 142 HR/min; RPE: 11.9; Velocity: different intensities.	The week prior to commencement of exercise. -Before, at half-time, immediately post-exercise. -6 h, 24 h, and 48 h post-exercise.	Mean saliva concentration of IgA was unaffected either by both intermittent and continuous exercise.	-	Soccer-specific intermittent exercise did not suppress the sIgA compared to continuous exercise (with the same overall work rate), although there with not significant differences founds.
Sari-Sarraf et al. [27]	10 males non-professionals	Yes	Two trials of soccer-specific intermittent exercise [24]: -Single session. -Double session.	-Single session at 14:30 h. -Double session in 1 day at 10:30 (1st training) and 14:30 (2nd training).	Intermittent exercise	-Before and immediately after exercise.	Single session: sIgA level increased significantly immediately post-exercise. -Double session: it does not appear a suppression of salivary IgA outcomes.	-	Two 90 min exercise sessions performed at a moderate intensity with a 2.25 h rest between them, do not necessarily have adverse effects on sIgA.
Sari-Sarraf et al. [28]	9 males amateur	Yes	2 trials of intermittent exercise 48 h apart on a motorized treadmill [24]	6 periods of 15 min. 15 min of rest between 3–4 periods.	Increasing exercise intensities (standing, walking, jogging, cruising and sprinting).	-Before 2 exercise trials. -Immediately after 2 exercise trials. -After 24 and 48 h following the 2 exercise trials.	-SIgA concentration was increased significantly immediately after both exercise trials and returned to pre-exercise levels 24 h later. -Although not statistically significant, there was a progressive decrease in resting SIgA concentration from exercise 1 to pre-exercise 2 (48 h after exercise 1) and to 48 h after exercise 2.	-	2 bouts of intermittent exercise with 48 h between sessions were not sufficiently stressful to induce compromises in s-IgA responses. The trend for a progressive reduction in resting sIgA observed 48 h after each exercise session has clinical relevance is unclear.
Page et al. [25]	10 male semi-professionals	Yes	Short-term soccer-specific fixture congestion [24].	Six × 15-min bouts of intermittent activity, with a 15-min rest between 3rd and 4th 3 bouts in 5 days with 48 h between them.	High intensity activity interspersed with periods of low intensity passive and activity recovery.	Two times: -at rest. -Immediately after each trial.	No significant main effects for time or trial, and no significant interactions were identified for the sIgA data.	-	A period of short-term fixture congestion did not show a significant cumulative or residual fatigue response across successive bouts.

HIIE: high-intensity interval exercise; HR: heart rate; IgA: Immunoglobulin A; RPE: respiratory perceived exertion; sIgA: salivary immunoglobulin A; URTI: upper respiratory tract infection; URTS: upper respiratory tract symptoms.

**Table 2:** Immunoglobulin A responses considering matches as a stressor during intensive or non-intensive fixture schedules.

Ref.	Sample	Stressor				Immunology (IgA)		URTS/URTI Relationship	Conclusions
		Int. Fix. Schedule	Test	Volume	Intensity	Salivary Test	Outcomes		
Moreira et al. [30]	24 male professionals	No	Friendly match	70 min	Non-official match.	Before and after the match.	A significant decrease in IgA protein were observed.	-	Participating in a 70-min regulation match does not significantly affect sIgA.
Mortatti et al. [9]	14 male U19 of Brazilian Championship	Yes	Congested match schedule	7 matches in 20 days	A progressive increment from 1 match to 7 match.	In the morning of each match day.	The decrements in sIgA, specially, in matches 2 and 6	Significant correlations were seen between the individual reports of URTI and the decrease in IgA levels in match 2 and 6.	The monitoring of sIgA could provide a useful and noninvasive approach for predicting URTI occurrences. In fact, decrements in mucosal immunity (IgA concentrations) may lead to a greater incidence of URTI.
Morgans et al. [32]	21 males English Premier League	Yes	Intensity winter fixture schedule	From match 1 to 5 in 15 days. From match 6 to 7, 1 match/week	-	2 days after each match prior breakfast	sIgA was significantly decreased after games 3, 4 and 5 when compared with game 1 After match 6–7 values were not different from game 1	-	A congested winter fixture schedule induces detectable perturbations to mucosal immunity. A decline in sIgA was evident in players that played >50% of the total minutes played.
Peñailillo et al. [33]	9 male professionals.	No	One international match.	Distance covered 9463 ± 458 m	-	Before and 10 min post-match.	IgA concentrations decreased by 74.5% post-match.	-	Decreased after match from pre-match salivary test. It seems that footballers with smaller decreases in testosterone levels covered more distance and decreased their immune function less.
Maya et al. [29]	16 female professionals from the Chilean 1st League.	Yes	Two final matches in 3 days.	2 final matches.	-	Before and after 2 matches (4 samples).	IgA concentration did not change after any match.	-	Salivary cortisol and testosterone concentrations increased especially after the first match of a final, without affecting IgA levels. It was speculated that increased testosterone concentration in women after football matches may play a protecting role against immune suppression usually observed after intense exercise.
Freitas et al. [5]	26 male young (15 years)	Yes	-One official match. -One simulated match 48 h after the 1st match.	35 min halves and 10 min rest.	Higher RPE for official match than non-official	10 min before the pre-match warm-up. Post-match saliva samples were collected within 10–15 min of the end of each match.	-	-	Official match may have led to a decrease in the main mucosal immunity function parameter that could increase the risk of URTI. Plan appropriate training loads and recovery procedures to avoid or minimize the likelihood of URTI occurrences should be considered.
Moreira et al. [31]	16 male young (14 years)	Yes	Congested match schedule	7 matches in 7 days 1 match = 20 min halves and 10 min rest	Days 1 and 2 = 2 match/day. Days 3, 4 and 5 = rest days. Day 6 = 2 matches. Day 7 = 1 match.	Before breakfast each morning with match day (i.e., 1, 2, 6, and 7), and on day 3 (1st rest day).	A significant change in sIgA concentration was observed across time points. A decrease in sIgA concentration was reported between the 1st and the 7th. The sIgA concentration at the rest day was higher in comparison with all other time points except the 1st time point.	-	Accumulated fatigue induced by a congested match schedule might negatively affect their mucosal immunity.

IgA: Immunoglobulin A; RPE: respiratory perceived exertion; sIgA: salivary immunoglobulin A; URTI: upper respiratory tract infection.

**Table 3:** Immunoglobulin A responses considering preparation periods (matches/training or training) as a stressor during intensive or non-intensive fixture schedules.

Ref.	Sample	Stressor				Immunology (IgA)		URTS/URTI Relationship	Conclusions
		Int. Fix. Schedule	Test	Volume	Intensity	Salivary Test	Outcomes		
Putlur et al. [13]	14 NCAA (Div. 3) female collegiate	No	9-week competitive season	9 weeks	-	At the beginning of each week	The levels of sIgA were suppressed throughout the course of the season.	82% of illnesses could be explained by a preceding decrease in sIgA.	Decreased levels of sIgA and increases in the indices of training load (load, strain and monotony) were associated with an increase in the incidence of illness.
Nakamura et al. [35]	12 males collegiate	No	A training period of 2 months	-	-	Daily	-	The saliva flow rate and sIgA secretion rate tended to decrease 3 days before the appearance of URTI symptoms compared to that in the non-infection period.	The findings suggest that monitoring of sIgA secretion rate may be useful for assessment of risk status of athletes for URTI.
Vardiman et al. [16]	12 female collegiate.	No	13 weeks during season	2 weeks with 2 training sessions followed by 1 rest day. Four sessions and 2 days competition. Each session: 86 min	-	Eight age-matched controls. Samples were collected bimonthly from the athletes’ pre-and post-sport training sessions and pre- and post-90-min sedentary period for the controls.	There was no significant difference between the athletes’ and controls’ mean difference pre- to post-sport training absolute sIgA levels.	Analysis of URTI total symptom per day indicated that there was no difference between the athletes and controls throughout the 13-week. There was no significant correlation found between URTI per day and absolute sIgA, URTI and Total protein, or URTI and absolute sIgA/Total protein levels.	The lack of relationship between sIgA levels and URTI indicate that sIgA is not an appropriate measure to determine an athlete’s susceptibility.
Fredericks [36]	24 males from EPL.	Yes	Training—training recovery	-	1st week after summer off-season. Pre-season training exercises.	-Before training. -20 min after training. -18 h after training.	It was a significant difference in sIgA across the pre, post 20-min and post 18 h periods	-	The changes in sIgA reflect the expected pattern of pressures and stresses associated with training and match-play. Overnight rest was enough to reverse a decrease in sIgA observed following a training session, but not following two consecutive matches played within a short period.
	9 males from EPL.	Yes	Match—Match recovery	53 h between matches. -1st half: 5.3 km -Whole match: 10.8 km.	Sample was a bottom team, and it plays against 1st and 2nd top teams.	-20 min after 1st match. -16 h after the 1st match. -20 min after 2nd match. -11 h after the 2nd match.	No significant difference between the 1st match post 20 min and the 1st match post 15h samples. No significant difference between the 20-min and 10 h post 2nd match.		
Moreira et al. [8]	26 male young (12 years)	No	21-week competitive season divided into preseason, competitive season, and detraining	Preseason trainings: 12 Competitive period´s sessions: 7 Recovery sessions: 2	-	Four time points: Before (T1) and after (T2) preseason, after the competitive phase (T3), and after the 2-week detraining phase (T4).	A significant increase in the sIgA rate was detected after the 2-week detraining period (T4), when compared with the value from T1.	URTI total symptom score was attenuated after the 2-week detraining period (T3–T4) when compared with T1–T2 and T2–T3.	Training and competition demand affect the mucosal immune responses. Adequate training periodization, including a short-detraining phase performed after accumulated training and competition periods might minimize undesirable outcomes such as mucosal immunosuppression and reduce URTI symptoms.
Morgans et al, [34]	13 male professionals from the preparation camp for the 2014 FIFA World cup	Yes	Microcycle (days -4, -3, -2, -1)	Four training sessions.	-	4 days preceding each game	sIgA displayed a progressive decline during the 4 days training period such that MD-1 values were significantly lower than both MD-4 and MD-3.	-	A short-term soccer-training camp in preparation for international competition induces detectable perturbations to mucosal immunity and thereby provides objective evidence for the administration of appropriate interventions to prepare players for the physical stress for the game day by self.
Owen et al. [3]	10 male professionals from European top team	No	Training sessions with different intensities.	Four training sessions.	-Low intensity session: tactical, technical. -High intensity sessions: conditioning sessions	-Before investigation (3 tests for baseline). -Before and after each training session.	Post-training sIgA were not different between high-intensity and low-intensity sessions at the first three periods. However, at the 4th session, SIgA concentration for high-intensity session was significantly lower than the low intensity session.	-	High-intensity sessions may cause a significant decrease in players sIgA values during the post-exercise window when compared against low-intensity training. The intensity and volume of the training sessions may be more appropriate to monitor in order to determine the impact of the IgA value. Coaches and practitioners are encouraged to monitor sIgA in routine, and in particular during high-intensity training periods so as to take precautions to avoid URTI in highly trained athletes.
Owen et al. [37]	37 male professionals.	Yes	Euro 2016 preparation period. Microcycle (days -4, -3, -2, -1)	-	Low, moderate, and high intensity sessions.	Pre-breakfast 90 min before training session.	Higher scores of sIgA were observed in MD-2 and MD-1, comparing to all others. No differences were found between playing positions with sIgA assessment.	-	A short-term preparation for international competition induces detectable perturbations in IgA.
Francavilla et al. [38]	35 male professionals (Italian Serie A)	No	A season.	Players played a higher number of minutes at t1 and t3 while they were substituted more frequently in the t2 and t3.	-	Three times: t1: after the pre-season period and 16 official matches played. t2: after a winter break and three official matches played. t3: 2 days after the final match of the championship and 19 matches played.	sIgA mean concentrations: t1: 122 t2: 131 t3: 147	-	No significant increase in the period characterized by a limited number of training sessions and of matches played. The weak increase in salivary IgA concentrations documented for the period of the season characterized by a limited number of training sessions, as compared to the periods of high-intensity training.
Figueiredo et al. [2]	18 male professionals from the preparation camp for the Rio 2016 Olympic Games.	Yes	Microcycle	Four training consecutive sessions.	TL increases from day 1 to day 2 but lowered from day 2 to day 3.	3 consecutive days before breakfast. Thus, the sIgA values reported represent day +1 of the preparation (each test assesses the effects of the previous training day).	-sIgA displayed a likely moderate decrease from day 1 to day 2 but increased on day 3.	-	sIgA can be used as an additional objective tool in monitoring football players. sIgA might be more responsive to training volume rather than intensity.
Lopes et al. [10]	U15: 17 players. U17: 22 players. U19: 18 players.		A season.	1.5 h/session and 70 (U15), 80 (U17) and 90 (U19) minute match per week.	-	Monthly, before the 1st session of the week (after at least, 36 h of rest).	The U19 team tended to show higher mean sIgA values than the younger teams.	There was a trend to younger players (<15 years) were more prone to get an URTS episode.	Monitoring salivary biomarkers provides information on mucosal immunity with impact in URTI symptoms occurrence. Players older than 15 years might have a stronger immune response possibly because of a natural sports selection process, which retains the more resilient athletes. Coaches could manipulate training loads to attenuate the physical stressors imposed on athletes, especially at demanding and stressful periods.

IgA: Immunoglobulin A; RPE: respiratory perceived exertion; sIgA: salivary immunoglobulin A; URTI: upper respiratory tract infection.
